# All-systolic non-ECG-gated stress perfusion CMR: improved visualization of subendocardial defects compared to conventional ECG-gated imaging

**DOI:** 10.1186/1532-429X-17-S1-Q122

**Published:** 2015-02-03

**Authors:** Behzad Sharif, Reza Arsanjani, Rohan Dharmakumar, Noel C Bairey Merz, Daniel S Berman, Debiao Li

**Affiliations:** 1 Biomedical Imaging Research Institute, Cedars-Sinai Medical Center, Los Angeles, CA, USA; 2 Cedars-Sinai Heart Institute, Los Angeles, CA, USA

## Background

The effects of ischemia are first realized in the subendocardium and progress transmurally. Hence, detection of the transmural extent of myocardial perfusion (MP) defects has important diagnostic/prognostic implications. Systolic MP imaging using ECG-gated methods has been shown to provide improved visualization of subendocardial defects [1]. We developed an innovative non-ECG-gated perfusion CMR technique capable of imaging all slices at the *same* systolic phase, and hypothesized that it improves visualization of the transmural extent of MP defects compared to the conventional method in CMR studies of CAD patients.

## Methods

A non-ECG-gated continuous acquisition scheme was developed using a steady-state FLASH sequence to achieve multi-slice T1-weighted imaging without saturation recovery (SR) preparation [2,3]. The sampling scheme used radial readouts acquired continuously in a slice-interleaved order (flip angle: 30°, TE: 1.4 ms, echo spacing: 2.7 ms, prescribed resolution: 1.4x1.4x10 mm). Retrospective systolic self-gating was performed automatically based on a low-resolution real-time reconstruction. The self-gated data was then used to perform high-resolution reconstruction of all slices in the same systolic phase using a compressed sensing approach with a data-adaptive sparsity constraint. A total of 30 MP studies at 3T were conducted in healthy subjects (n=5) and patients (n=10) with suspected CAD (based on prior abnormal nuclear MP study) using the proposed all-systolic method (1^st^ day visit) and the conventional ECG-gated SR-prepared method (2^nd^ day visit).

## Results

**Figure 1:**
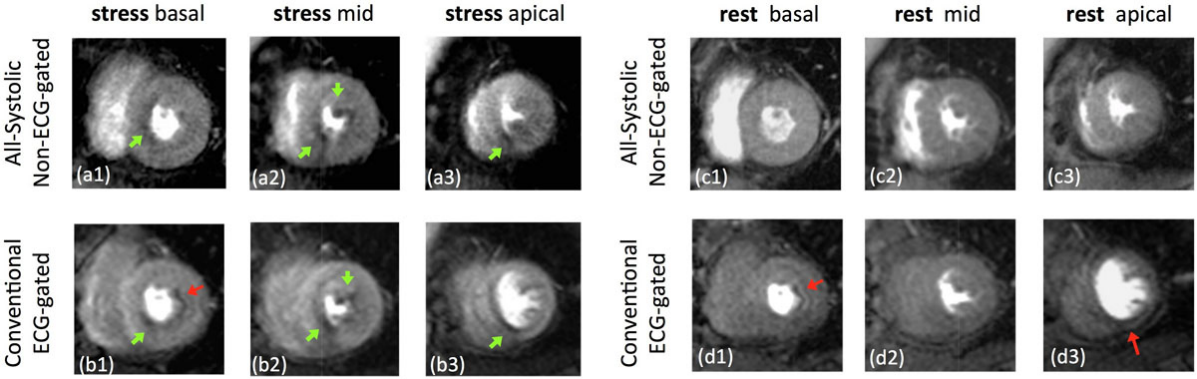
Representative adenosine stress/rest patient study. (a,b): stress myocardial perfusion study using the proposed non-ECG-gated all-systolic method (first-day visit) and the conventional ECG-gated method (second-day visit); (c,d): rest myocardial perfusion studies corresponding to each of the two methods for the same patient. Red arrows point to subendocardial dark-rim artifacts (LGE scan was negative). The stress-induced MP defects (green arrows) were closely matched between the two methods. However, the apical slice for the conventional scan was imaged at the diastolic phase; this combined with the presence of dark-rim artifacts reduces the reliability of detecting the transmural extent/severity of stress perfusion defects for the conventional method. In contrast, all of the perfusion defects for the proposed method were imaged at the end-systolic phase, enabling reliable determination of their transmural extent.

The all-systolic MP studies in healthy subjects demonstrated normal perfusion, no dark-rim artifacts, and similar myocardial contrast-to-noise ratio (8.6±0.6) compared with the conventional scheme (8.0±0.7). A representative stress/rest patient study is shown in Fig. 1. Among all patients who had an abnormal CMR study (n=8), the detected stress MP defects matched between the two methods (after ruling out artifacts seen on conventional MP scans based on rest/LGE scans and patient history; 2 expert readers in consensus). Of all the observed defects on stress MP images, 26% were at or near end-diastole in the acquired conventional MP images, making it nearly impossible to visualize their transmural extent. In contrast, all of the perfusion defects for the proposed method were imaged at the end-systolic phase, enabling reliable determination of their transmural extent (Fig. 1).

## Conclusions

Conventional perfusion CMR methods require accurate ECG gating and do not provide the freedom to image all slices at systole. As indicated in the presented results, the proposed non-ECG-gated all-systolic perfusion CMR method has the advantage of improved visualization of subendocardial defects and thereby potentially improved reliability compared to conventional approaches or other ungated methods.

## Funding

American Heart Association Scientist Development Grant 14SDG20480123; National Institutes of Health NHLBI grant nos. K99 HL124323-01, R01 HL038698-18, R01 HL091989-05, R01 HL090957-01.
